# Ética en el uso de psicodélicos: la definición de las drogas ilícitas bajo la óptica de la bioética crítica

**DOI:** 10.18294/sc.2024.4630

**Published:** 2024-02-14

**Authors:** Sergio Alexandre Liblik, Thiago Rocha da Cunha, Carmem Silvia da Fonseca Kummer Liblik, Diego Nicolás Biscioni, Dennys Robson Girardi

**Affiliations:** 1 Magíster en Bioética. Profesor, Pontifícia Universidade Católica do Paraná, Curitiba, Brasil. aliblik@gmail.com Pontifícia Universidade Católica do Paraná Pontifícia Universidade Católica do Paraná Curitiba Brazil aliblik@gmail.com; 2 Doctor en Bioética. Profesor, Programa de Pós-grado em Bioética, Pontifícia Universidade Católica do Paraná, Curitiba, Brasil. caixadothiago@gmail.com Pontifícia Universidade Católica do Paraná Pontifícia Universidade Católica do Paraná Curitiba Brazil caixadothiago@gmail.com; 3 Doctora en Históra. Investigadora, Grupo de Pesquisa CNPq/PUCPR Bioética, Saúde Global e Direitos Humanos, Pontifícia Universidade Católica do Paraná, Curitiba, Brasil. carmemsfk@gmail.com Pontifícia Universidade Católica do Paraná Pontifícia Universidade Católica do Paraná Curitiba Brazil carmemsfk@gmail.com; 4 Magíster en Salud Pública. Profesor, Departamento de Salud y Actividad Física, Universidad Nacional de Avellaneda, Avellaneda, Argentina. diegobiscioni@gmail.com Universidad Nacional de Avellaneda Universidad Nacional de Avellaneda Avellaneda Argentina diegobiscioni@gmail.com; 5 Magíster en Tecnología en Salud. Doctorando em Direito Empresarial e Cidadania, Centro Universitário Curitiba, Curitiba, Brasil. dennys.girardi@live.com Centro Universitário Curitiba Centro Universitário Curitiba Curitiba Brazil dennys.girardi@live.com

**Keywords:** Psicodélicos, Interdicción Legal, Salud Colectiva, Bioética, Derechos Humanos, Psychedelics, Legal Interdiction, Public Health, Bioethics, Human Rights

## Abstract

Este ensayo, ubicado en el campo de la bioética, analiza la prohibición del uso de psicodélicos, explorando argumentos sobre las crecientes evidencias de potenciales terapéuticos y su historia milenaria de usos culturales y espirituales. Discute inicialmente el contexto histórico de los psicodélicos y diferentes términos utilizados para nombrarlos. Problematiza la definición de “drogas”, indicando falta de criterios objetivos para distinción entre lícitas e ilícitas. Bajo conceptos y referenciales teóricos de la bioética crítica, se analiza cómo el discurso moral prohibicionista se sostiene más por intereses políticos y económicos que por justificativas científicas, generando estigmatización y vulnerabilidad. Defiende el fin de la prohibición de los psicodélicos con base en argumentos éticos, resaltando su importancia para la reducción de sufrimientos individuales y colectivos. El trabajo contribuye a una reflexión profundizada sobre este tema socialmente controversial, articulando conocimientos interdisciplinarios.

## Abstract (en)

This essay, situated in the field of bioethics, examines the prohibition of psychedelic use, exploring arguments surrounding the growing evidence of their therapeutic potential and their millennia-long history of cultural and spiritual uses. It initially discusses the historical context of psychedelics and the various terms used to describe them. The essay problematizes the definition of “drugs,” highlighting the lack of objective criteria for distinguishing between legal and illicit substances. Drawing on concepts and theoretical frameworks of critical bioethics, it analyzes how the prohibitionist moral discourse is sustained more by political and economic interests than by scientific justifications, leading to stigmatization and vulnerability. The essay advocates for the end of the prohibition of psychedelics based on ethical arguments, emphasizing their importance in reducing individual and collective suffering. The work contributes to a deeper reflection on this socially controversial topic, integrating interdisciplinary knowledge.

## INTRODUCCIÓN

El uso de sustancias psicoactivas con propiedades visionarias o enteogénicas se remonta a los albores de la humanidad. Hallazgos arqueológicos sugieren la presencia de psicodélicos en rituales religiosos y prácticas comunitarias ya en el periodo Neolítico, hace aproximadamente 10 mil años^1^. En la medicina tradicional china e hindú también se empleaban plantas psicoactivas por sus efectos psíquicos. Sustancias con efectos psicodélicos han sido encontradas en prácticas tradicionales y restringidas del budismo tibetano^2^. 

En occidente, referencias al uso de sustancias como el opio, el cáñamo y el vino se remontan a la antigüedad clásica^3^. A lo largo de milenios, pueblos originarios de América utilizaron enteógenos como la ayahuasca, el peyote y los hongos psilocibinos en sus prácticas espirituales y terapéuticas^4^. Estudios interdisciplinarios en el área de la farmacología, biología y antropología sugieren que la inclusión incidental de psicodélicos en la dieta de los homínidos y su eventual adición a rituales e instituciones de los primeros humanos podría haber conferido ventajas selectivas^5^^,^^6^. 

La psilocibina y psicodélicos similares que apuntan principalmente al subtipo de receptor de serotonina 5-hidroxitriptamina (5-HT) estimularían una respuesta de estrategia de afrontamiento activa, que puede haber proporcionado una capacidad mejorada para cambios adaptativos a través de un modo de cognición flexible y asociativo, ya que, en aquel contexto, los efectos de los psicodélicos podrían haber aumentado la sociabilidad, la imaginación, la elocuencia y la sugestionabilidad de los primeros pueblos^6^.

A partir del siglo XIX, el aislamiento de los principios activos de diversas plantas da inicio a la era de los psicodélicos sintéticos, culminando con la síntesis de la dietilamida de ácido lisérgico (LSD) en la década de 1930. Por dos décadas, investigadores indagaron el potencial terapéutico de los psicodélicos en más de miles de pacientes^7^. 

Sin embargo, en la segunda mitad del siglo XX, durante la Guerra Fría, bajo la influencia de EEUU, y con un raro ejemplo de consenso con el bloque soviético, casi la totalidad de los países adoptó, por incentivo de la Organización de las Naciones Unidas (ONU), leyes prohibicionistas, enmarcando a los psicodélicos como “drogas” sin valor médico y con alto potencial de abuso^8^. Este radical cambio de estatus, en descompás con milenios de uso, aún carece de explicación convincente frente a las evidencias históricas y actuales. Pero, como señala Escohotado^9^, no debe ser desdeñado que el acuerdo prohibicionista sobre el LSD (por las siglas en alemán de *Lysergsäure-diethylamid*) y otros psicodélicos, durante la Guerra Fría, favoreció el monopolio compartido de las industrias de psicofármacos estadounidenses y soviéticos. 

En las últimas décadas del siglo XXI, ha ocurrido un resurgimiento del interés científico en el estudio de las sustancias psicodélicas, fenómeno que viene siendo llamado “renacimiento psicodélico”^10^. Este renovado entusiasmo contrasta con el estatus de prohibición y criminalización que recae sobre el uso y la investigación con estas sustancias en la mayoría de los países. 

En Latinoamérica, aunque no existen legislaciones específicas sobre psicodélicos, algunos países están adoptando enfoques de mayor apertura respecto a las drogas. Avances legislativos en Uruguay (Ley 19172 del 11 de diciembre de 2013), Colombia (Ley 1787, del 2 de julio de 2016), Bolivia (Ley 906, del 8 de marzo de 2017) y Argentina (Ley 27350, del 19 de abril de 2017) han llevado a la descriminalización del uso medicinal y terapéutico del cannabis, con algunas restricciones relacionadas con los derivados y la concentración^11^.

A pesar de las restricciones legales, diversas instituciones de investigación fueron creadas recientemente con el objetivo específico de investigar el potencial terapéutico y los mecanismos de acción de ese grupo de sustancias^12^. Por lo tanto, se configura actualmente un escenario de antagonismo, en que la prohibición y criminalización conviven con un florecimiento de investigaciones que buscan rescatar el valor medicinal y espiritual que los psicodélicos poseyeron en diversas culturas a lo largo de la historia de la humanidad.

Recientemente, en el contexto de Latinoamérica, Martínez Oró *et al*.^13^^)^ y Suárez y Clua-García^14^^)^ presentaron en la revista *Salud Colectiva* importantes análisis críticos sobre la lógica prohibicionista con relación a drogas como cannabis y psicodélicos, mostrando cómo este proceso es una agenda político-moral ajena, que va en contra de la defensa de la salud pública y de los derechos humanos en la región. 

En consonancia, con esta perspectiva, este ensayo, desarrollado desde una mirada teórica de la bioética crítica^15^^,^^16^^,^^17^^,^^18^^,^^19^, una aproximación latinoamericana de la bioética basada en un diálogo entre la *teoría crítica* de la escuela de Frankfurt y los *estudios decoloniales*, amplía y profundiza el debate ético sobre los usos asistidos con psicodélicos, analizando la definición de drogas ilícitas, los argumentos sobre la legitimidad de las políticas prohibicionistas bajo las evidencias que vienen siendo producidas sobre sus usos terapéuticos, tanto como de los impactos de las guerras a las drogas y su relación con dimensiones sociales y morales de la vulnerabilidad.

## LOS PSICODÉLICOS Y SUS NOMBRES

Los psicodélicos son conocidos por diversos nombres, lo que refleja visiones distintas sobre esas sustancias desde los diferentes campos del conocimiento. El término “psicodélicos” tiene un problema de doble sentido entre sustantivo (clase de fármacos) y adjetivo (efecto subjetivo atribuido al uso). La traducción del inglés “*psychedelics*” a “psicodélicos” en español y portugués, posiblemente refuerza la idea errónea de correlación del prefijo “psico” con la psicosis. Por eso, también se encuentra en estos idiomas la expresión “psiquedélicos”. Por el mismo motivo, también se encuentra la expresión “enteógenos” como forma de destacar experiencias en usos tradicionales, místicos y espirituales.

En este trabajo, optamos por el término “psicodélicos” cuando nos referimos a los estudios científicos y “enteógenos” cuando necesitamos resaltar sus aspectos tradicionales o espirituales. Desde el punto de vista ético, ninguna de esas formas de uso puede ser indicada, *a priori*, como más legítima o adecuada que otra. De todos modos, se reconoce que la multiplicidad de otros nombres (psicomiméticos, alucinógenos, psicodislépticos, etc.) refleja las incongruencias entre puntos de vista de los diversos campos del conocimiento sobre psicodélicos. Cada nombre representa una preconcepción sobre los efectos de esas sustancias.

Por encima de todo, es importante destacar que el concepto común de “drogas” no tiene sustento científico consensuado, siendo simplificador y moralizador del debate. Un estudio acerca de las representaciones sociales del concepto de “drogas” indicó que nombrarlas de este modo generalmente atribuye significado previo de nocividad, incluso de demonización o pánico moral^20^, lo que, además de ocultar los efectos nocivos de tantos otros productos farmacéuticos que causan toxicidad, dependencia o mismo “alucinaciones”, también estigmatiza el conocimiento sobre el potencial terapéutico, cultural y espiritual de muchos psicodélicos.

Por regla general, los psicodélicos se dividen en dos grandes grupos: los clásicos y los atípicos. En el caso de los psicodélicos clásicos, también conocidos como típicos, sus moléculas se presentan de manera similar a la serotonina, actuando especialmente en receptores de las células neuronales del tipo 5-HT2A. Entre ellos se encuentran la dietilamida del ácido lisérgico (LSD); la mescalina, presente en cactus como el peyote y el San Pedro; la dimetiltriptamina (DMT), encontrado en la ayahuasca; y la psilocibina, sustancia presente en los hongos “mágicos”^21^.

 Se sabe que el receptor 5-HT2A no es el único objetivo de estas sustancias; sin embargo, es su activación la que media los efectos psicodélicos. Con relación a los psicodélicos atípicos, están presentes sustancias con diversos mecanismos de acción. Algunos autores prefieren subdividir los atípicos en cuatro subgrupos: los disociativos (ketamina y fenciclidina), los empatógenos (MDMA), los canabinoides (cannabis) y los propiamente atípicos (salvinorina, ibogaína, beleño, borrachero)^21^.

La clasificación de drogas psicodélicas es realizada por investigadores que intentan agruparlas conforme a determinados criterios. Si, por un lado, hay consenso en agrupar los psicodélicos clásicos y atípicos conforme a sus propiedades, por otro, no hay concordancia con el gran grupo de sustancias atípicas presentado. Es decir, algunos autores clasifican los canabinoides como un grupo aparte y aún el MDMA como una sustancia estimulante, junto con la cocaína y las anfetaminas. 

Algunos psicodélicos atípicos, con excepción del cannabis, presentan toxicidad y pueden llevar al deceso dependiendo de la dosis utilizada, como la ibogaína y los anticolinérgicos^22^. Mientras que los psicodélicos clásicos -como la mescalina, el LSD, el DMT y la psilocibina- son considerados sustancias con bajo riesgo, principalmente porque no producen grandes alteraciones cardiorrespiratorias, no generan uso compulsivo o abstinencia y son débiles las evidencias de que induzcan dependencia^7^^,^^10^^,^^21^. Estas características contribuyen para que los psicodélicos sean cada vez más estudiados en contextos de investigación clínica de forma ética y segura^9^^,^^10^^,^^11^^,^^12^, explorando sus efectos subjetivos y potenciales terapéuticos.

### Evidencias de usos terapéuticos de los psicodélicos

El aumento epidémico de los diagnósticos de enfermedades asociadas a la salud mental ocurre incluso en países donde hubo aumento del acceso a los tratamientos psiquiátricos y psicoterapéuticos clásicos^23^. Preocupantemente, esa epidemia ocurre en concomitancia a una disminución en las inversiones en las investigaciones, en el número de patentes y en el desarrollo de nuevos medicamentos en el área de la salud mental^24^, sugiriendo que las ya insuficientes herramientas terapéuticas no poseen perspectiva de proporcionar soluciones adecuadas para el problema^25^^,^^26^^,^^27^. 

Asociadas a la posibilidad de que los compuestos psicodélicos vistos no solo como una innovadora clase medicamentosa, sino como parte de un nuevo paradigma de tratamiento, la psicoterapia asociada a los psicodélicos (PAP)^28^, podría modificar esa tendencia, trayendo una justificación ética contra la prohibición y las resistencias institucionales a los usos asistidos de psicodélicos.

Los psicodélicos pueden ser definidos como agentes farmacológicos que son capaces de, activando innumerables vías fisiológicas, provocar alteraciones en la percepción, induciendo estados no-ordinarios de consciencia, así como provocar alteraciones en los afectos y en la cognición. Por otro lado, con relación al potencial de los psicodélicos para el uso en el tratamiento del abuso de sustancias, es bien conocido. Por ejemplo, en el tratamiento del alcoholismo^29^, de la dependencia de tabaco^30^ y en la dependencia de opioides^31^.

En los últimos años, ensayos doble ciego controlados indican que tanto el LSD, como la metilendioximetanfetamina (MDMA) y la psilocibina fueron capaces de reducir sufrimientos relacionados con la ansiedad^32^, el dolor^33^, la depresión^34^, entre innumerables condiciones de salud mental^35^^).^ Publicaciones de estudios realizados en Latinoamérica, especialmente con ayahuasca, también han mostrado iguales potenciales de usos terapéuticos^36^^,^^37^.

Entre las diversas investigaciones, se destacan estudios que caracterizan los efectos promotores de neuroplasticidad asociados a estas sustancias, un fenómeno con gran importancia teórico-práctica en diversos campos de la ciencia biomédica. Ly *et al*.^38^, por ejemplo, muestran que compuestos psicodélicos como el LSD, la N,N-dimetiltriptamina (DMT) y la 2,5-dimetoxi-4-iodoanfetamin (DOI) aumentan la complejidad del árbol dendrítico, promueven el crecimiento de la columna dendrítica y estimulan la formación de sinapsis. Esos efectos celulares, como veremos, destacan el potencial de los psicodélicos para el tratamiento de la depresión y los trastornos relacionados, así como también dentro del gran árbol de las enfermedades degenerativas neurológicas.

En relación con los riesgos fisiológicos, aunque en el contexto de los psicodélicos parecen ser menos significativos en comparación con otros psicofármacos^39^, en los casos de los psicodélicos clásicos existe una preocupación relacionada con los riesgos cardiovasculares y, en particular, con la aparición de picos hipertensivos durante las sesiones de uso, descritos como de intensidad leve a moderada en algunos sujetos^40^. Por otro lado, los riesgos psíquicos no pueden ser totalmente descartados^41^, sobre todo con relación a experiencias intensamente negativas y sufrimiento emocional continuo, especialmente en aquellos con historia personal o familiar de esquizofrenia o trastorno bipolar^42^. Además, es posible considerar que los riesgos de los psicodélicos en el ambiente terapéutico podrían ser mayores que cuando son usados en contextos religiosos o incluso recreativos^43^. Esto ya es conocido desde los trabajos pioneros de Anthony Wallace en las décadas de 1940 y 1950, y el problema referente al contexto del uso (*set and setting*) es uno de los paradigmas generalmente más aceptados^44^.

Aun así, incluso si la presencia de episodios de ansiedad (disforia aguda) o de un estado de psicosis transitoria, descrito como agudo, en las experiencias puede tener un significado diferente y más benigno de lo que la comprensión actual de la psiquiatría considera^45^, es cierto que los terapeutas que deseen trabajar con los psicodélicos necesitan tener una conciencia anticipada y realista de estos riesgos.

Considerando la evaluación de riesgos y beneficios, Nichols^45^ agrupa detalladamente las principales áreas terapéuticas que podrían beneficiarse con los psicodélicos, en las que se incluyen: 1) alivio de la ansiedad y depresión de pacientes con enfermedades que amenazan la vida o terminales (o sea, dentro del espectro de cuidados paliativos); 2) trastorno de humor unipolar; 3) trastorno obsesivo-compulsivo; 4) tratamiento de dependencia química (alcohol, nicotina, opioides); 5) cefalea en racimos; 6) trastorno del espectro autista; 7) glaucoma (como hipotensores); 8) como nootrópicos, en la mejora en la función cognitiva; 9) o de la creatividad; 10) en la regeneración tisular; 11) por sus efectos en la respuesta inmune (incluso postulándose sus efectos en enfermedades alérgicas y autoinmunes); y 12) en la diferenciación y crecimiento celular.

Esas investigaciones vienen siendo acompañadas por una modificación de la opinión pública de forma favorable^46^, asociándose a la creación de centros de investigación específicos^47^^)^ y en un momento en que la revisión de las prohibiciones legales pasa a ser una exigencia de especialistas del campo^48^.

De esta forma, frente al estado del arte sobre las potenciales acciones terapéuticas de los psicodélicos, y considerando la determinación de sus riesgos como, *a priori*, no más importantes que los riesgos asociados a la farmacología psiquiátrica tradicional, se justifica éticamente un abordaje abierto, no-estigmatizante y no-restrictivo de los usos asistidos de psicodélicos.

### Droga, finalmente, ¿qué es eso?

Diferentes estudios han señalado que el paradigma prohibicionista de las drogas no se fundamenta únicamente en criterios de salud, sino en una red de intereses económicos, especialmente vinculados al crimen organizado que subsiste a través del comercio ilícito, y en intereses políticos, dirigidos principalmente al control social de grupos marginados, como las juventudes de las periferias y las comunidades tradicionales^49^^,^^50^. De acuerdo con Martínez Oró *et al*.^13^, al considerar los parámetros prohibicionistas para el cannabis y otros psicodélicos, queda nítido que el criterio para tornar ilícitas a determinadas drogas, no son sus riesgos objetivos, sino una agenda política y moral que es sostenida por intereses económicos e ideológicos. Es decir, lo que diferencia las drogas “lícitas” de las drogas “ilícitas” es pura y simplemente la propia prohibición, lo que vuelve la definición tautológica. 

La Oficina de las Naciones Unidas contra la Droga y el Delito (UNODC) aborda “droga” como un término variado que, en el campo de la medicina, se refiere normalmente a las sustancias que previenen, curan o mejoran la salud. En la farmacología, incluye cualquier sustancia química que modifica los procesos fisiológicos y bioquímicos. Ya en el ámbito de la fiscalización internacional, retomando convenciones datadas de 1961 y 1971, la UNODC define como “drogas” las sustancias con potencial de adicción, alto riesgo a la salud pública y bajo o nulo valor terapéutico, incluyendo, entre ellas, los psicodélicos^8^.

Al analizar esta última definición, comúnmente usada en los debates prohibicionistas, queda aún más evidente la ausencia de sustentación objetiva para la definición de drogas. Por ejemplo, si las drogas son sustancias que causan dependencia, entonces el alcohol o el tabaco, entre tantas otras sustancias, deberían ser prohibidas, principalmente si consideramos los daños a la salud pública provocados por su uso^51^, así como sus nulos efectos terapéuticos. Curiosamente, en este caso, no deberíamos prohibir los psicodélicos clásicos, pues la gran mayoría de los usuarios de alucinógenos no pasa a situaciones dependencia^52^^,^^53^.

Si el criterio fuera la toxicidad, lo que llevaría a una preocupación de salud pública, tal definición tampoco encontraría base científica^45^. De hecho, incluso en contextos de usos no asistidos, los efectos tóxicos de los psicodélicos permanecen raros^54^. En EEUU, por ejemplo, la incidencia del tratamiento médico de emergencia buscado por psicodélicos es más baja comparada a cualquier otra droga de usos recreativos, con 1,9 atenciones de emergencia identificados cada 1.000 en 2011. En relación con las internaciones, la tasa de “alucinógenos” como sustancia primaria es de 0,1% de las internaciones^54^. 

Esa situación se vuelve más paradójica cuando se considera la hipótesis de que los psicodélicos, con su relativa baja toxicidad y dependencia, son importantes como respuesta no solo de tratamientos individuales, sino de salud pública relacionada con los abusos de opioides y de otras adicciones^26^^,^^27^^,^^28^. Estas constataciones sobre los posibles efectos positivos en el ámbito de la salud pública serían, incluso, uno de los principales argumentos objetivos para retirar los psicodélicos de las listas de drogas ilícitas de la UNODC, si no fuera esta una decisión puramente política y moral.

El único criterio que restaría para sostener la prohibición de los psicodélicos sería, en verdad, un punto particularmente controversial de las justificativas prohibicionistas, que se refiere al “riesgo psicológico”, desencadenamiento de “psicosis” o de “alucinaciones persistentes”. Sin embargo, a pesar de las leyendas urbanas y de los innumerables mitos construidos en torno al tema, la identificación de estos fenómenos no encuentra sustentación en la literatura, estando restringida a pocos relatos de casos^54^.

Al respecto, es importante distinguir entre estados de alucinaciones involuntarias, con pérdida de la comprensión de la realidad y desfragmentación, con los estados llamados “holotrópicos”^55^_._ En ese estado, estas experiencias son descritas como un estado no-ordinario de la conciencia, un estado alterado de la consciencia o un estado amplificado del inconsciente, relatadas como “trascendentes”, “místicas”, “de carácter numinoso”, observándose patrones que son diferentes a los observados en psicosis^55^.

Como ya hemos abordado, uno de los problemas centrales en este análisis se resume en la nominación de los psicodélicos, muchas veces identificados como “alucinógenos”. De la misma forma que las alteraciones sensoriales no pueden ser igualadas a la psicosis, tampoco se puede afirmar que la fenomenología experimentada es un efecto alucinatorio de las drogas, aún más sabiendo que esa misma fenomenología puede ser provocada a través de simples prácticas corporales, como la respiración holotrópica^56^ o, incluso, pueden ser obtenidos a través de técnicas milenarias, como diversas tradiciones de místicos, yoguis o meditadores^57^. Por lo tanto, el hecho de prohibir los psicodélicos porque inducen estados no-ordinarios de percepción de la realidad tampoco se sostendría si los argumentos fuesen científicos.

Finalmente, una de las alegaciones siempre traídas a la discusión se refiere al hecho de que habría un sostenimiento de la opinión pública para justificar el estado prohibicionista de las “drogas”. En muchos países, la opinión de que las drogas deben ser prohibidas es casi unánime.

En Brasil, por ejemplo, en una encuesta con 2.400 personas, la mayoría de los participantes se manifestó a favor del mantenimiento de la prohibición de la venta y del consumo de crac (94%), cocaína (94%) y marihuana (80%). No se realizó ninguna pregunta específica sobre psicodélicos típicos; sin embargo, cuando se les preguntó sobre qué grupos deberían tener más peso en la definición de leyes y políticas sobre drogas en general, el 81% de los participantes indicaron médicos y psicólogos y el 51% mencionaron jueces y policías, reforzando un abordaje punitivita y patologizante sobre el tema^58^. Incluso, las propuestas de usos asistidos de drogas, como el establecimiento de locales seguros y bajo supervisión médica como estrategia para reducir daños en el uso de opioides en EEUU, tienen apenas un 29% de la aprobación de la población adulta en aquel país^59^.

Es cierto que no se podría esperar resultados diferentes en relación con la opinión colectiva, principalmente después de tantos años de publicidad antidrogas en todo el mundo. Hay que considerar, al respecto, que la falta de conocimiento histórico impide a la opinión pública utilizar los ejemplos previos de este tipo de estrategia política, como el caso de EEUU en los años de la Ley Seca, que fue relacionada con el aumento importante de la violencia. Además, como las drogas son muchas -y el mensaje creado por la ideología prohibicionista las iguala-, las personas simplifican todo bajo el signo de “tóxicos” o “venenos”.

De esta forma, al contrario de ser tomada en consideración como razón para mantener la prohibición de los psicodélicos, los impactos de la opinión pública deben ser considerados críticamente en cuanto a su impacto en procesos de estigmatización y vulneración. En el citado estudio de opinión pública realizado en Brasil, la palabra “droga” fue asociada a términos como destrucción, ruina, degradación personal o familiar, error, muerte, bandidaje, entre otros conceptos estigmatizantes^58^. En otro estudio sobre representaciones sociales de las drogas, el consumo fue caracterizado como práctica “inmoral”, “peligrosa” o como forma “hedonista” de obtener placer^20^. 

Frente al problema del estigma social, Suárez y Clua-García^14^, con quienes concordamos, abogan por un cambio en las políticas públicas represivas hacia modelos amparados por ley que incluyan la autorregulación del uso de las drogas por los propios usuarios. Esta sería una forma no solo de reducir las estigmatización y exclusión de las personas que utilizan drogas como psicodélicos, sino también sería una forma activa de afirmar valores como el respeto a la dignidad humana y la libre determinación.

### Usos de psicodélicos bajo perspectivas de la bioética crítica

La discusión sobre los motivos por la cual los psicodélicos siguen prohibidos en gran parte del mundo, así como sobre sus efectos negativos en relación con sujetos, grupos y la colectividad, exige una mirada interdisciplinaria pautada en diálogos no jerarquizados entre ciencias biomédicas, ciencias sociales, humanas y saberes no-científicos. 

El campo de la bioética fue propuesto justamente como un “puente” entre los diversos involucrados en los macro y micro conflictos éticos que envuelven la vida humana y no humana^60^. En Latinoamérica, una formulación llamada bioética crítica^15^^,^^16^^,^^17^^,^^18^ fundamenta su propuesta teórica y metodológica a partir de sus referentes de la teoría crítica de Frankfurt y en los estudios decoloniales, lo que permite un análisis profundizado de conflictos éticos de naturaleza global, como es el caso de la prohibición de los psicodélicos.

Estos referenciales permiten develar, por ejemplo, que la agenda prohibicionista se mantiene por una razón instrumental de matriz moderna y colonial que sigue conformando los valores que son asumidos por la opinión pública, científicos, legisladores, jueces y profesionales de la salud involucrados directa o indirectamente en los debates sobre usos de drogas ilícitas. 

La instrumentalización de la razón prohibicionista queda evidente, sobre todo, cuando se consideran las contradicciones sobre la forma de tratamiento de determinadas drogas “ilícitas”, como los psicodélicos, frente a otras drogas “lícitas” que son altamente rentables para las grandes industrias capitalistas, como el alcohol, el tabaco y los opioides. 

El aspecto colonial de la razón prohibicionista se revela, a su vez, al constatar cómo sus discursos buscan categorizar de modo negativo tanto a los “usuarios de drogas” de los centros urbanos como a las diversas prácticas culturales y espirituales que hacen usos chamánicos de psicodélicos como ayahuasca, peyote u hongos.

Además de las lecturas sobre razón instrumental y colonialidad, la bioética crítica provee claves para comprender cómo discursos morales son muchas veces utilizados para legitimar lo opuesto de aquello que constituiría axiológicamente los propios valores. Cunha^61^, por ejemplo, analizando críticamente el proceso de constitución de las agendas de salud global en los Objetivos de Desarrollo Sustentable (ODS) de la ONU, muestra cómo valores relacionados con los derechos humanos, como dignidad, solidaridad y equidad, son utilizados como forma de legitimar políticas de salud enfocadas en coberturas mínimas de servicios proporcionados por empresas privadas que, al contrario de garantizar aquellos valores, los anulan. 

Situación parecida se encuentra en las directrices internacionales de la UNODC^8^ que, al justificar el prohibicionismo a partir del mismo campo axiológico de los derechos humanos, tiene resultados que operan lo opuesto: la generación de una guerra a las drogas marcada por la violación sistemática de derechos humanos con fuerte recorte de persecución étnico-racial^62^, resultando en forma de “colonialidad de la vida”, o sea, del “proceso de creación de una ontología de la vida que autoriza a pensar que unas vidas son más importantes que otras, desde el punto de vista político, fundando así una jerarquía y justificación para la dominación, la explotación y el sometimiento”^63^.

En el ámbito de la teoría crítica en las relaciones internacionales, Robert Cox^64^ presenta el concepto de “gran nebulosa” para caracterizar la forma de gobernanza difusa de la economía política globalizada, marcada por articulaciones de intereses explícitos e implícitos tanto en agendas públicas como en reuniones informales de clubes y cofradías formadas por intersecciones de intereses de corporaciones financieras e industriales, agendas religiosas y facciones criminales que mantienen, en el contexto de un orden mundial arbitrario, los límites de actuación de los propios Estados nacionales^64^. 

Esa forma “nebulosa” de gobernanza parece aplicarse también a la estructura del prohibicionismo global, en la medida en que se alinea, por medio del sostenimiento normativo de la “guerra a las drogas”, a un verdadero complejo militar-médico-financiero-industrial que lucra tanto con las guerras como con los monopolios que se derivan de la oposición arbitraria de “drogas lícitas e ilícitas”. Estudios más profundizados, a partir de estos referenciales, pueden auxiliar a identificar, en la articulación también difusa de los grupos que siguen resistiendo al prohibicionismo, la formación de una “contra-nebulosa de la guerra a las drogas”. 

De todo lo que fue expuesto en este trabajo, queda claro que el prohibicionismo es un discurso moralista, con sus dualismos sobre bien/mal, bueno/malo, correcto/incorrecto. Ese discurso moral se sostiene en supuestos saberes médicos, jurídicos y políticos que asocian las “drogas” al mal, y no afectan solo a las personas usuarias, sino también a quienes investigan la sustancia, así como a profesionales que prescriben los psicodélicos con fines terapéuticos, volviéndolos, en potencia, “moralmente vulnerables”. 

El principio de la vulnerabilidad moral, también elaborado en el campo de la bioética latinoamericana^19^, llama la atención sobre una dimensión específica de vulnerabilidad que es esencial para la comprensión del problema en discusión, en la medida en que resalta los sufrimientos causados a ciertos grupos o individuos que sufren discriminación y estigmatización por no compartir valores de la moralidad hegemónica, contándose aquí los múltiples aspectos que contribuyen a la formación de tal moralidad como, por ejemplo, aspectos ideológicos, culturales, religiosos, filosóficos y científicos, lo que es precisamente el caso aquí analizado. 

La prohibición de los psicodélicos puede ser reconocida como éticamente problemática aún bajo diversos otros aspectos, incluso por interferir en la libertad y la autodeterminación de las personas sobre sus modos de vida, así como en práctica espiritual y cultural de diversos grupos que hacen uso de psicodélicos alrededor del mundo. De este modo, por coherencia, no hay por qué proponer en este análisis formas de jerarquización del uso de drogas conforme a alguna tabla moral, una vez que eso incurriría en aquello que denuncia el propio principio de la vulnerabilidad moral.

Así, al defender el fin de la prohibición de los usos de psicodélicos, estamos admitiendo que desde un punto de vista ético toda forma de uso es *a priori* lícita, siendo que debería importar para el individuo y la sociedad solo si el uso está siendo nocivo o alienante como, por ejemplo, bajo la perspectiva de la adicción. Por otro lado, no debemos aceptar indiscriminadamente la rotulación de recreativo para el uso de las sustancias, principalmente cuando se nombra de esta forma en oposición al uso terapéutico y refrendado por la ciencia, en una narrativa que no esconde una moralidad selectiva y jerárquica.

## CONSIDERACIONES FINALES

Pasados 50 años de su prohibición global, los psicodélicos y su historia ancestral no desaparecieron. Por el contrario, su interés, en oposición a su estatus de prohibición, nunca disminuyó, principalmente en el uso “ilegal” de millones de personas en todo el planeta que continuaron haciéndolo, ahora bajo pena de inmensas sanciones legales, y sin la orientación o la seguridad que solo pueden ser viabilizados adecuadamente en contextos no prohibitivos. 

Podemos verificar que las restricciones a los psicodélicos no se sostienen por justificativas científicas, clínicas o sanitarias, sino por una sobreposición de intereses políticos y económicos que determinan -y son dialécticamente determinados- por un discurso moralista que vulnerabiliza, estigmatiza y excluye. 

Al respecto, uno de los problemas centrales adoptados en el trabajo fue la propia nomenclatura negativa de las “drogas” como representación social para abordar los psicodélicos, que conforme fue presentado, no constituye solo una cuestión semántica, sino figura como un violento dispositivo moral. 

Se verificó, también, que el paradigma moral prohibicionista sostiene, normativamente, un poderoso complejo militar-médico-financiero-industrial que, por medio de una gran nebulosa de intereses, mantiene una violenta y lucrativa guerra a las drogas que, en el fondo, es una guerra a las personas y grupos que las utilizan.

De la misma forma que en las últimas décadas, las macrocuestiones éticas como el racismo, el feminismo y la orientación sexual precisaron ser combatidas inicialmente desde una posición de ilegalidad (visto que la oposición a esos derechos era respaldada por las leyes y fortalecida por la moral entonces hegemónica), actualmente es preciso enfatizar las diferencias entre legitimidad y legalidad y defender una ética de los usos de psicodélicos.

Así, postulamos que el fin de la prohibición de los psicodélicos debe ser uno de los objetivos de una práctica crítica de la bioética orientada a la reducción del sufrimiento, individual y colectivo, tanto en la dimensión microética, cuando el foco es el análisis de los posibles usos terapéuticos de los psicodélicos, como macroética, cuando contextualizamos la prohibición de los psicodélicos en la larga cadena de intereses que sostiene la guerra a las drogas.
